# Screening of Dietary Ingredients against the Honey Bee Parasite *Nosema ceranae*

**DOI:** 10.3390/pathogens10091117

**Published:** 2021-09-01

**Authors:** Chiara Braglia, Daniele Alberoni, Martin Pablo Porrini, Paula Melisa Garrido, Loredana Baffoni, Diana Di Gioia

**Affiliations:** 1 Department of Agricultural and Food Sciences (DISTAL), University of Bologna, Viale Fanin 44, 40127 Bologna, Italy; chiara.braglia4@unibo.it (C.B.); loredana.baffoni@unibo.it (L.B.); diana.digioia@unibo.it (D.D.G.); 2 Instituto de Investigaciones en Producción Sanidad y Ambiente (IIPROSAM), Centro Científico Tecnológico Mar del Plata-CONICET-UNMdP-CIC-PBA, Funes 3350, Mar del Plata Zc 7600, Argentina; mporrini@mdp.edu.ar (M.P.P.); pmgarrid@mdp.edu.ar (P.M.G.); 3 Centro de Investigación en Abejas Sociales (CIAS), FCEyN, UNMdP, Funes 3350, Mar del Plata Zc 7600, Argentina

**Keywords:** nosemosis, *Vairimorpha ceranae*, nisin, *Saccharomyces* sp., acetic acid, para-coumaric acid, gut microbiota

## Abstract

*Nosema ceranae* is a major pathogen in the beekeeping sector, responsible for nosemosis. This disease is hard to manage since its symptomatology is masked until a strong collapse of the colony population occurs. Conversely, no medicaments are available in the market to counteract nosemosis, and only a few feed additives, with claimed antifungal action, are available. New solutions are strongly required, especially based on natural methods alternative to veterinary drugs that might develop resistance or strongly pollute honey bees and the environment. This study aims at investigating the nosemosis antiparasitic potential of some plant extracts, microbial fermentation products, organic acids, food chain waste products, bacteriocins, and fungi. Honey bees were singularly infected with 5 × 10${}^{4}$ freshly prepared *N. ceranae* spores, reared in cages and fed ad libitum with sugar syrup solution containing the active ingredient. *N. ceranae* in the gut of honey bees was estimated using qPCR. The results showed that some of the ingredients administered, such as acetic acid at high concentration, p-coumaric acid, and *Saccharomyces* sp. strain KIA1, were effective in the control of nosemosis. On the other hand, wine acetic acid strongly increased the *N. ceranae* amount. This study investigates the possibility of using compounds such as organic acids or biological agents including those at the base of the circular economy, i.e., wine waste production, in order to improve honeybee health.

## 1. Introduction

*Nosema ceranae* is a unicellular sporogenous fungus belonging to the phylum microsporidia, which gives rise to a chronic debilitating infection in honey bees named nosemosis [1]. This pathogen co-evolved with *Apis cerana*, whose parasitism became endemic in Asia. Nevertheless, *Apis mellifera* colonies infected by *N. ceranae* were found for the first time in 2005 in Taiwan [2] and in 2006 in most European countries [3]. When this host species shift occurred is unknown, although if it is reasonable to believe that it happened at the time of *A. mellifera* introduction in Asia, in the 1880s [4]. The exact *N. ceranae* arrival period in Europe is not clear but evidence suggests that it has been present in Europe since 1998 [5], thanks to an active international trading of *A. mellifera* from Asia to the rest of the world. Recently, a revision and redefinition of the genera Nosema and Vairimorpha proposes to rename *N. ceranae* and *N. apis* as *Vairimorpha ceranae* and *Vairimorpha apis* [6] which, more than taxonomic consequences, could become relevant for future research in the topic. Adult honey bees easily become infected by ingesting spores from stored honey and pollen and subsequent transmission through trophallaxis [7,8], also after the exposure to surfaces contaminated by spores, following the colony cleaning and visits to contaminated flowers and pollen in the foraging activity [7,9]. After the ingestion, microsporidia spores germinate extracellularly in the midgut lumen and then inject the sporoplasm in an epithelial cell through the polar tube [1]. Infected colonies at the beginning show no visible symptoms and when environmental conditions are favorable for the parasite, they may rapidly collapse [10], making nosemosis a disease that sometimes is hard to control and difficult to diagnose and cure. The mycotoxin fumagillin (dicyclohexylamine salt), produced by *Aspergillus fumigatus*, is the first and the only successful antibiotic for the treatment of nosemosis [11,12,13] since 1953 [14]. Currently it is available on the market in many American countries and Korea, but it is forbidden in the European Union because of the absence of a detailed threshold residue regulation in honey and hive products [15]. Fumagillin use is nowadays controversial: targeting the methionine aminopeptidase-2 (MetAP2) [16], an enzyme present in many eukaryotes, can cause metabolic imbalances also in non-target organisms, i.e., it has been classified as mutagenic and cytotoxic for mammals after a short-term exposure [17]. Fumagillin residues persist in hives and its degradation products pose a potential risk for human health [18,19]. For instance, fumagillin toxicity was assessed in honey bees, causing a reduction in their lifespans [20], an alteration of structural and metabolic proteins in midgut [21], a reduction in sperm quality [22] and health [13]. Moreover, its efficacy depends on several factors such as seasonality and plantations [23]. Furthermore, in laboratory conditions, Huang et al., 2013 [21] have demonstrated that mature *N. ceranae* spore proliferation was similar in treated and untreated bees at the recommended fumagillin concentration (250 µg/L) and induced *N. ceranae* hyper proliferation when fumagillin concentration was 10 folds lower. Therefore, the need to find new strategies, which combine honey bee health protection and governmental standards in terms of food safety, gave a new pulse to the research of alternative solutions. Studies aimed at verifying the effect of veterinary drugs or commercial dietary supplements on the honey bee gut microbiota composition have been published recently. In recent years [24,25], researchers have begun to evaluate the use of plant extracts as *Nosema* control agents, such as thymol, oregano oil, carvacrol, cinnamaldehyde, resveratrol, and garlic based products, and treated bees showed lower *N. ceranae* infection rates compared to control [26,27,28]. Moreover, *Andrographis paniculate*, *Cryptocarya alba*, *Gevuina avellane*, *Artemisia dubia*, and *Laurus nobilis* extracts were tested with positive results [29,30,31,32,33], supporting a plant extract based strategy as a promising tool in the control of bee diseases. Brassicaceae seeds also showed promising results for their protective effects against N. ceranae spores at the laboratory level [34]. Furthermore, the use of beneficial microorganisms like *Bifidobacterium* and *Lactobacillus* strains [35,36], and *Bacillus subtilis* metabolites [37,38] showed encouraging results. For example, Baffoni et al. (2016) [35] demonstrated that probiotic treatment with *Lactobacillus* and *Bifidobacterium* strains reduced the presence of *Nosema* spores in naturally infected bees, thus proving the efficacy of a preventive microorganism-based strategy. Commercial probiotic preparation also based on lactic acid bacteria were found to be effective against the same parasite [27,36]. Similarly, De Piano et al. [39] demonstrated a strong relation between bacterial metabolites and the count of *N. ceranae* spores, showing a significant decrease after *Lactobacillus johnsonii* AJ5 administration. Additionally, organic acids produced by lactic acid bacteria present in the honey bee’s environment (flowers, beebread, and gut), such as lactic acid, acetic acid, and phenyl-lactic acid, were tested, through feeding, against these microsporidia and showed a strong reduction in spore load in bees [38]. Therefore, these compounds have particular interest for the beekeeping sector. The aim of the present study was to test feed ingredients belonging to 4 different groups (organic acids, *Saccharomyces* and antibiotics, wine derivatives, and plants extracts) for their antimicrobial activity against *N. ceranae* in *A. mellifera* workers, but also to evaluate their toxicity on individual honey bees.

## 2. Materials and Methods

### 2.1. Experimental Set up

Cage experiments were carried out in the microbiology laboratory of the Department of Agricultural and Food Sciences, University of Bologna, in the period between October 2019 and June 2021. Newly emerged honey bees (*Apis mellifera ligustica* were obtained from multiple brood frames of emerging honey bees within the University of Bologna experimental apiary (San Lazzaro, Bologna, Italy, 210 m a.s.l.) in a continental climate. At least two independent laboratory cage tests were performed for each feed ingredient in order to validate results. The first set of assays, referred to as “First screening”, was organized in order to study the effects of the feed ingredients on honey bee survival and parasite development. After this first screening, a second one, referred to as “Derived tests”, was performed in order to confirm the obtained results or to test new hypotheses derived from the first one. Moreover, if the results showed a promising trend, even if not significant, the dosage of the active ingredient was increased, determining two different dosages for some of the ingredients [AA, NisA and GRA] marked as “low” (_L) and “high” (_H). Only para-coumaric acid and acetic acid were tested a third time using summer honey bees due to the contrasting results obtained in the two tests. All the tests were performed with the same protocol, having only differences in the season at which the newly emerged honey bees were obtained. The ingredients assayed were chosen considering their known antimicrobial properties in both food and feed safety (organic acids and antibiotics), their immune stimulation properties (mainly plant extracts and microorganisms), but also basing the choice on traditional homemade remedies used by beekeepers (e.g., wine derivatives) that do not have a proper scientific basis. The selected compounds reported in Table 1 and are divided in four main groups: i. organic acids (acetic acid [AA], abscisic acid [ABA], p-coumaric acid [pCA]); ii. wine derivatives (ethanol [EtOH], sulphites [SUL], wine vinegar [WA]); iii. *Saccharomyces* and antibiotics (*Saccharomyces* sp. strain KIA1 [SC], a mixture of gramicidin A, B, C, and D [GRA], Nisine A [NisA]); iv. plant extracts (extract of *Opuntia ficus-indica* [OPT], extract of brown alga *Padina pavonica* [PP], a mixture of manuka and tea tree oil [MT]). In all the tests, fumagillin [DCH] was used as positive control. Each assay had a dedicated control test, i.e., a group of infected bees not supplemented with any compounds in their diet [CTR]. Experimental cages had a dimension of 11 × 7 × 4 cm and were made using plastic, including a ventilation mesh. Three replicate cages, containing 50 newly emerged honey bees, were prepared for every dietary treatment and controls. Mortality in every replicate cage was registered on a daily basis, extracting the dead individuals after counting. Ten worker bees for each experimental condition were sacrificed at day 9 (experiment end) and the guts (midgut and rectum) were collected individually and stored at −20 °C until tissue analysis.

### 2.2. Production of N. ceranae Spores

Honey bee colonies infected with *N. ceranae* were identified in a apiary nearby Modena (Italy), in the city of Savignano sul Panaro (44°29′03.4″ N 11°03′28.1″ E). Spores were collected from diseased hives by capturing flying foragers honey bees over the colony entrance. Obtained foragers were sacrificed and the midgut and rectum extracted and broken up in distilled water. Spores were divided in multiple stocks and conserved in a 10% glycerol PBS solution at −80 °C to guarantee the same *N. ceranae* strain availability for all the assays. When an assay was established, spores were retrieved from cryostat and about thirty-five newly emerged bees were caged and infected by feeding bees with the prepared solution to allow *N. ceranae* proliferation and sporulation, in order to obtain fresh and highly infective spores. A sample of the spores obtained was characterized and confirmed as *Nosema ceranae* according to [44] and the same stock was used for all infections. Fresh spores were extracted from infected and caged honey bees, counted with Neubauer chamber to estimate the load per ml, diluted in a sugar syrup solution to the final concentration of 10${}^{4}$ spore/mL and used as a fresh and standardized *N. ceranae* propagules for the ongoing assay.

### 2.3. Oral Infection with N. ceranae Spores

Newly emerged honey bees (*Apis mellifera ligustica*) were obtained from brood frames with worker bees ready to emerge. Brood frames were picked from three different colonies for every assay, in order to homogenize the genetic variability [45]. Frames were were maintained under controlled conditions (32 °C; 60% RH) until honey bees emerged. Then, newly emerged bees were kept in ventilated cages for 2 days until individual inoculation with control treatment (syrup) or 5 × 10${}^{4}$ *N. ceranae* freshly prepared spores in syrup according to Porrini et al. [46]. The temperature and relative humidity were maintained during the experiments at 29 °C and 60% RH, respectively. Before starting the administration of the ingredients, a sample of newly emerged individuals was taken to test the potential basal infection with *N. ceranae*.

### 2.4. Cultivation of Saccharomyces sp.

*Saccharomyces* sp. was isolated from forest soil collected at the Bologna Apennines (Italy) 250 m a.s.l. (data not shown) and it was grown on PDB culture broth and incubated at 120 rpm for 5 days at 35 °C. Then, the obtained culture was mixed in sugar syrup 1:1 (*w*:*v*) in equal amounts in order to obtain 10${}^{11}$ CFUmL.

### 2.5. Production of Plant Extracts

*Padina pavonica* seaweeds were collected from the Mediterranean sea during summer 2019, by hand picking method from the submerged marine rocks. The seaweeds were cleaned from impurities, dried, and powdered with a mixer. The algal flour thus obtained was extracted sequentially with hot sodium oxalate solution, hot water, 1 M and 4 M KOH solution at 20 °C according to [47] and resuspended in glycerol. The *Opuntia ficus-indica* hydroglycolic extract was obtained from fresh cladodes collected in the Malta Island during summer 2019, which were washed with distilled water and cut into small pieces before extraction. A 20:80 (*w*/*w*) water/propylene glycol mixture was used with a 1:3 (*w*/*w*) plant-solvent ratio. The cladodes were macerated for 12 h and the resultant mixture was percolated to obtain the extract [48].

### 2.6. Treatment Administration

Ingredients were administered in sucrose syrup (1 kg sucrose in 1 L of water) using gravity feeders starting from a day after the artificial infection with *N. ceranae* spores. The active ingredients and concentrations for each dietary treatment administered in a matrix of sucrose syrup are reported in Table 1. The tested ingredients (treatments) were supplied to honeybees a day after the artificial infection with *N. ceranae*. The treatments were available *ad libitum* during 9 days. Honey bees in every experimental cage also received tap water *ad libitum* in gravity feeders.

### 2.7. DNA Extraction and qPCR of N. ceranae

Extracted gut were manually macerated with plastic micro pestles in 200 µL of buffer. DNA extraction of single honey bee guts was performed with PureLink${}^{TM}$ Genomic DNA Mini Kit (Invitrogen, Milan, Italy) following the manufacturer protocol with some modifications: guts were further smashed with glass beads (0.01–0.1 µm) at 50 Hz in Rotovortex. Moreover, samples were incubated at 55 °C in a water bath with 180 µL of lysis buffer and 20 µL of proteinase K per sample [49]. Fluorometric quantification of every sample was performed with Qubit Flex Fluorometer (Thermo Fisher Scientific). Extracted DNA was stored at −20 °C until further analysis. The 16S-like rRNA gene (SSU rRNA) was selected to perform *N. ceranae* specific qPCR relaying on specific primer Nc841f 5′-GAGAGAACGGTTTTTTGTTTGAGA-3′ and Nc980r 5′-ATCCTTTCCTTCCTACACTGA TTG-3′ [50]. The reactions were carried out on Step One thermal cycler (Applied Biosystems) with standard two-step PCR method using Fast SYBR Green PCR Master Mix (Life Technologies, Milan, Italy), according to [51,51]. The standard PCR fragment was diluted 1:10 to obtain the reference standards. The melting curve was performed in each real-time reaction to assess amplicons melting temperature (73.77 ± 0.23 °C St. Dev) according to the genetic variability of *N. ceranae*. According to Cilia et al. [52,53], the copy number of 16S-like rRNA gene in *N. ceranae* ranges from 5.7 to 11.5 per genome, therefore the obtained quantification was normalized according with the total amount of extracted DNA and divided by the average copy number of 16S-like rRNA (i.e., 8.6).

### 2.8. Statistical Analysis

Obtained data were divided according to groups of ingredients, as described above; each assay had its own control, therefore obtaining different datasets. Datasets were analyzed with the R software [54], tested for normality and homoscedasticity with Shapiro and Levene’s tests. The dataset was corrected with Cooks Distance multivariate method, to identify outliers based on regression analysis comparison [55]. Datasets were analyzed with ANOVA when data were normal and homoscedastic, while a generalized linear model was applied for non-normal homoscedastic data. Bonferroni *p*-value correction for multiple comparisons was applied for every assay. Boxplots were generated with ggpubr and ggplot2 packages. *Nosema ceranae* infection load was expressed as Log *N. ceranae* units, considering the absolute quantification corrected for the average copy number as described above. To calculate, plot and compare the survival rates for every assay, daily mortality on each replicate cage was recorded and analyzed by means of Kaplan–Meier survival analysis and log-rank tests (SigmaStat Software, San Jose, CA, USA), which estimates also the median survival time.

## 3. Results

### 3.1. Survival Tests

**Figure 1:**
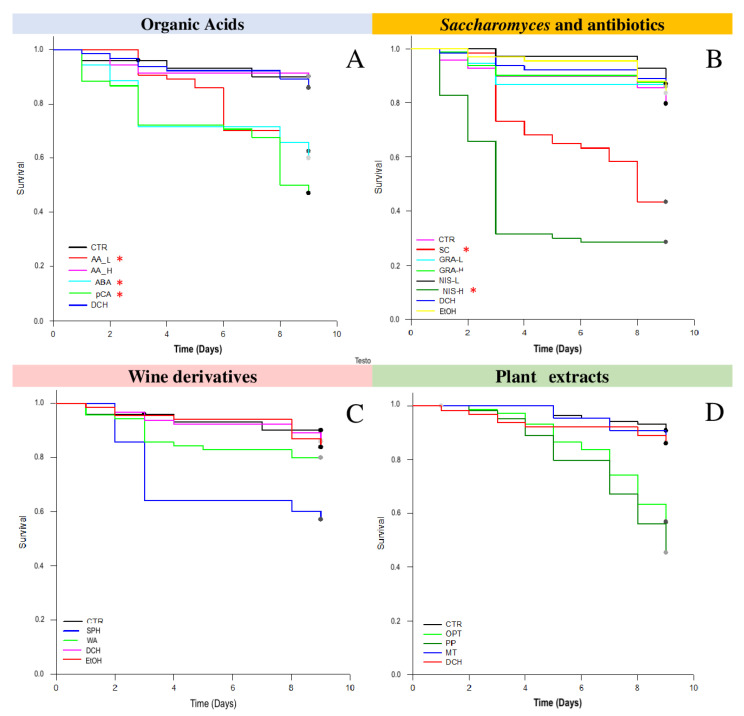
Survival of honey bees. (**A**) Survival curves for treatments in the test on “organic acids”; (**B**) Survival curves for treatments in the “test on microbial origin compounds”; (**C**) Survival curves for treatments in the test on “wine derivatives”; (**D**) Survival curves for treatments in the “plant extracts”; Asterisk (*) indicate significant differences with control treatment (Kaplan–Meier survival analysis, log-rank test; Statistical details for each test: 1A (*p* < 0.001, 52.256, df = 5); 1B (*p* < 0.001, 25.167, df = 7); 1C (*p* < 0.001, 176.870, df = 3) and 1D (*p* < 0.001, 51.352, df = 3). [AA_L] Acetic Acid lower concentration; Acetic Acid higher concentration [AA_H]; *p*-Coumaric Acid [pCA]; Abscisic Acid [ABA]; *Saccharomyces* sp. [SC]; a mixture of gramicidin A, B, C, and D [GRA]; Nisin higher concentration [NisA_H]; Nisin lower concentration [NisA_L]; Fumagillin [DCH]; infected control without treatments [CTR]. [*] *p* < 0.001).

Survival results of the test using organic acids, detailed in Figure 1A, showed a clear toxic effect of p-coumaric acid [pCA], abscisic acid [ABA] and the highest concentration of acetic acid [AA_H] compared with control treatment (*p* < 0.001). On the other hand, acetic acid [AA_L] at the lowest concentration (four times lower than [AA_H]) kept the survival response at the same level of the control treatment [CTR] and fumagillin [DCH]. The administration of *S. cerevisiae* live cells caused a progressive toxic effect to *N. ceranae* infected bees, reaching less than 50% of live bees at day 9 post-infection (Figure 1B). When bacteriocins were administered, a toxic effect was only detected for the highest dose of nisin [NIS_H]. The remaining treatments did not show significant changes in honey bees’ survival. Toxicity curves shown in (Figure 1C), related to wine-derived products, demonstrated no toxicity of wine vinegar [WV] with control treatment and fumagillin [DCH]. On the contrary, sulphites caused significant toxicity in bees (*p* < 0.001). The two plant extracts assayed [OPT and PP] caused a toxicity level higher than CTR and DCH, as detailed in (Figure 1D).

### 3.2. N. ceranae Quantification

In collected honey bees, natural infection of *N. ceranae* was predominantly absent, indeed the highest detection did not exceed 2.0 Log NcU.

#### 3.2.1. Acetic Acid and p-Coumaric Acid Decrease *N. ceranae* Units Only in Winter Honeybees

**Figure 2:**
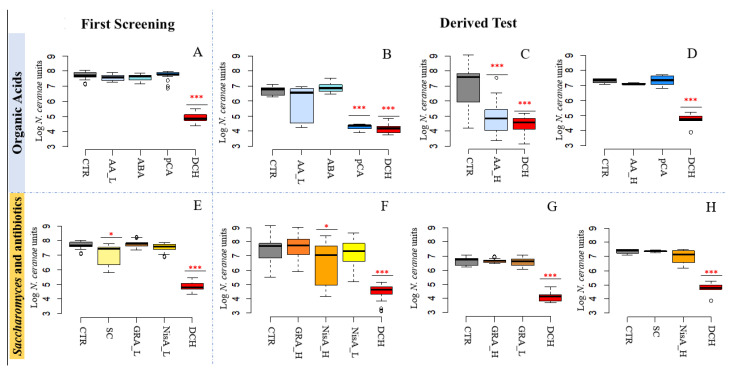
*N. ceranae* inhibition assays. Box plots from the first test (exploratory) and derived tests (confirmatory) are reporting the Log of *N. ceranae* units (NcU) per honey bee gut obtained at 9 days post inoculation with spores for every treatment with dietary ingredient. (I) results obtained from organic acids; (II) results obtained from *Saccharomyces* and antibiotics; Acetic Acid lower concentration [AA_L];Acetic Acid higher concentration [AA_H]; p-Coumaric Acid [pCA]; Abscisic Acid [ABA]; *Saccharomyces* sp. [SC]; a mixture of (**A**,**B**,**C**,**D**) [GRA]; Nisin higher concentration [NisA_H]; Nisin lower concentration [NisA_L]; Fumagillin [DCH]; infected control without treatments [CTR]. [*] *p* < 0.05; [***] *p* < 0.01.; boxplots (**B**,**C**,**F**) shows results obtained with winter honey bees; boxplots (**A**,**D**,**E**,**G**,**H**) shows results obtained with summer honey bees. Organic Acids: First Screening (**A**); Derived Test (**B**–**D**). *Saccharomyces* and antibiotics: First Screening (**E**); Derived Test (**F**–**H**).

In the first screening, after 9 days, CTR samples reached an infection rate of 7.69 ± 0.24 Log of *N. ceranae* units (NcU). On the other hand, the positive control fumagillin [DCH], significantly decreased the NcU reaching 4.11 ± 0.32 and 4.86 ± 0.30 Log NcU (*p* < 0.01), respectively. Treatments based on “organic acids” [AA, ABA, pCA] did not show any significant variation with respect to the CTR (Figure 2A). In the derived test, pCA showed a significant reduction in NcU, to [DCH] (Figure 2B, *p* < 0.01), but again this was not confirmed in a third test performed ad hoc (Figure 2D). Acetic acid at the lowest dose confirmed its inefficacy in the derived test, whereas at the highest dose [AA_H] it showed contrasting results in performed tests (Figure 2C,D).

#### 3.2.2. Nisin Has a Potential Effect on *N. ceranae*

In the test of “*Saccharomyces* and antibiotics” after 9 days of ingredients consumption CTR samples reached an infection rate of 6.64 ± 0.30 Log of *N. ceranae* units (NcU), respectively. A significant reduction was found for the treatments including *S. cerevisiae* [SC] (Figure 2E, *p* < 0.05) while nisin [NisA] showed a non-significant reduction in NcU with respect to the CTR. NisA at high dose showed a significant decrease (*p* < 0.05) which was not confirmed after repeating the test (Figure 2F,H). Finally, GRA confirmed the inefficacy at both high and low doses (Figure 2E–G).

#### 3.2.3. Wine Derivatives and Plant Extracts Do Not Reduce *N. ceranae* Parasite Development Treatments

**Figure 3:**
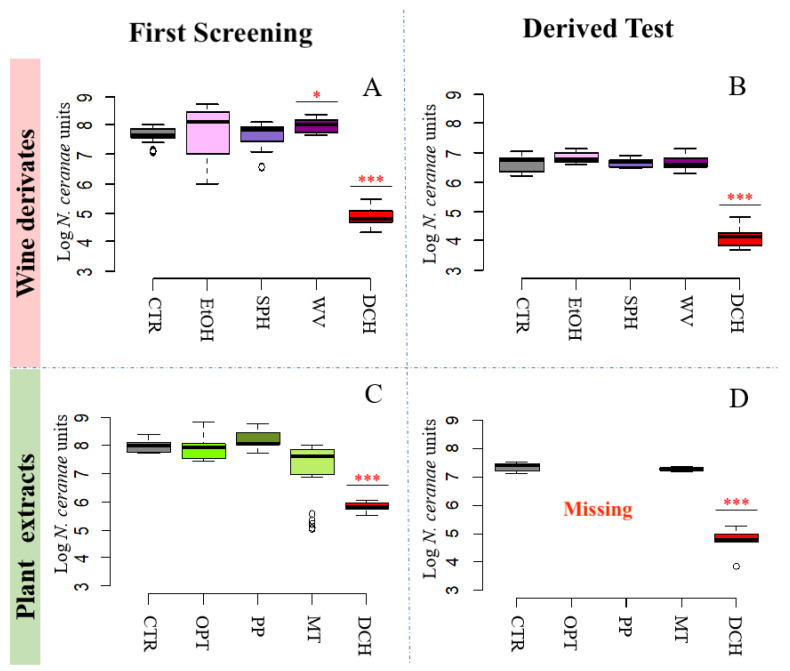
*N. ceranae* inhibition assays. Box plots from the first test (exploratory) and derived tests (confirmatory) are reporting the Log of *N. ceranae* units (NcU) per honey bee gut obtained at 9 days post inoculation with spores for every treatment with dietary ingredients. (I) results obtained from wine derivatives; (II) results obtained from plants extracts. Treatments included: Etanol [EtOH]; sulphites [SPH]; wine vinegar [WV]; *Opuntia ficus-indica* [OPT]; Padina pavonica [PP]; Manuka and Tea oil mixture [MT]; Fumagillin [DCH]; infected control without treatments [CTR]. [*] *p* < 0.05; [***] *p* < 0.01. Wine derivates: First Screening (**A**); Derived Test (**B**). Plant Extracts: First Screening (**C**); Derived Test (**D**).

In the test including “wine derivatives”, a significant increase was found for the treatment with wine vinegar [WV] in the first test up to 7.99 ± 0.24 Log of NcU (*p* < 0.05) with respect to the control [CTR] (Figure 3A,B), whereas the other substances tested [SPH and EtOH] did not cause significant differences, also in the derived test. None of the “plant extracts” tested [OPT, PP, and MT] showed significant changes, even if the mixture of Manuka and Tea oils [MT] showed a decreasing trend in the first test (7.12 ± 1.06 Log of NcU in [MT] vs. 7.69 ± 0.24 Log of NcU in [CTR]—Figure 3C,D). In the derived test OPT and PP caused the death of all honey bees until day 9 due to toxicity of the compounds used. All obtained data are reported in Table 2.

## 4. Discussion

This work investigated the efficacy of different organic and biological agents against *N. ceranae* with the aim of finding dietary ingredients or medicaments potentially usable to control nosemosis. In recent years, this topic has become a priority in honey bee research since, at present, there are no effective and safe treatments commercially available. It is worth noting that some of the ingredients were tested against *Nosema* for the first time, although some of them are commonly handled in beekeeping practice to fight pathogens.

### 4.1. Organic Acids and Wine Derivatives

Feeding honey bee colonies on sugar syrup supplemented with acidifying substances was associated with an improved development of the honey bee colonies during the active season [56,57]. Moreover, it is not unusual that beekeepers add acetic acid or other acidic products to the winter food [58,59,60]. The justification for this practice relies on the assumption that this additive aids digestion, reduces granulation in syrup, diminishes robbing [61], and prevents the formation of molds in feeders. Moreover, the acidity may also have effects on *Nosema* spore germination. Nevertheless, there are contradictory results on the impact of acidified food on *Nosema* infections, as some studies have indicated it has no impact on the development of nosemosis caused by *Nosema apis* [62]. In spite of this, in the present research, a *N. ceranae* reduction at the highest concentration of acetic acid was shown. However, in winter bees, the reduction was significant in comparison with summer honey bees, suggesting a seasonal dependence of the described effect. Indeed, the first test was carried out in autumn, whereas the derived tests were carried out using summer honey bees. A possible explanation of the different effect is that in temperate climates worker honey bees can either develop into short-lived summer bees or long-lived winter bees. The latter show effective overall immune response [63], besides having a different protein metabolism [64] and a different composition of the microbiota [65] in order to adapt to cold temperatures. All these factors may influence *N. ceranae* development in comparison with newly emerged honey bees obtained in the summer. Further investigations are needed to establish the efficiency of acetic acid on early summer honey bees, also in field conditions. Acetic acid has a low toxicity to insects based on available data with an LD50 > 50 µg/honey bee in an acute contact toxicity study [66]. It is worth noting that the lowest concentration of the acid caused a significant mortality, whereas the highest one was well tolerated by treated honey bees. Those findings are possibly explained by differences in the amount of food consumed, related with cumulative toxic effects derived from the chronic exposure or deterrent effects. Although this hypothesis should be tested also in a longer exposure condition, our findings might be promissory to propose this compound as a candidate for in-field applications. This is also consistent with a previous research in which the administration of *Lactobacillus johnsonii* CRL1647 produced lower spore counts possibly due to the production of acidic metabolites including lactic acid, phenyl-lactic acid, and acetic acid at a concentration of 38 mM [37]. Wine vinegar, which is mainly composed of acetic acid and traditionally used by beekeepers to prevent *N. ceranae*, in our research induced an unexpected increase in the spore load. The different results between the use of acetic acid and vinegar may be explained by the presence in wine vinegar of other secondary metabolites like hydroxymethylfurfural (HMF) that is toxic for honey bees [58,67,68,69], although, in this study, the mortality was only 20% at the end point. In the study conducted by Ptaszyńska and collaborators [70], a strong correlation between the concentration of ethanol to feed honey bees and the proliferation of *Nosema* spp. infection was observed but, also, a higher toxic effect when 5% and 10% ethanol under a chronic administration design was given. In our study, we supplied 0.69 M (4%) of ethanol in the diet, a concentration sufficiently high to generate cellular stress in honey bees according to [71], but not enough to cause differences on mortality rates or on *N. ceranae* development, in agreement with [70]. Furthermore, Ptaszyńska and collaborators [70] suggested a correlation between acidification derived from ethanol metabolism and *Nosema* spp. development based on a facilitation of the spore germination, which could also explain some of our results.

In our research, *p*-coumaric acid was used as it was reported to possess antimicrobial potential against a bacterial infection affecting honey bee larvae, *Paenibacillus larvae* [72]. At the dose of 31.4 µM (31 ppm), the phytochemical induced only a slight reduction in the microsporidia amount in the first test, whereas in the derivative test the reduction was highly significant when compared to the respective control, but, again, this result was not confirmed when the acid was tested a third time. As mentioned above, these contrasting results might be explained by a different honey bee physiology correlated with the season and foraging resources. Summer and winter bees show distinct physiology: long-lived winter bees (diutinus stage) are characterized by oxidative stress tolerance and longevity [73,74] and senescence is almost negligible [75,76,77], whereas in summer worker bees, there is rapid senescence after the nurse-forager transition. In this study, *p*-coumaric acid reduced *Nosema* development only in winter honey bees. This is only partially in agreement with the results obtained by Bernklau et al. [78] that showed a significant reduction in *N. ceranae* spores with a concentration of 25 ppm in summer honey bees. Although abscisic acid dietary supplementation has been reported to stimulate the immune response and host health in honey bees [79], as well as to possibly influence the nosemosis prevalence under field condition [41], the administration of the phytohormone did not affect the development of the infection and, in addition, it was found to be toxic for honey bees. Therefore, further studies are necessary to elucidate the effect of this molecule on the bee physiology in relation to the environment.

Sulphite compounds are often used in agriculture to counteract fungal diseases, such as powdery mildew (*Uncinola necator*) in grapevine cultivation. They are also widely used in the wine industry for wine clearance and the obtained organic sulphites are a waste with potential antimicrobial activity. The honey bee food supplementation with sulphites resulted inactive against the microsporidia *N. ceranae*. This is in contrast with [80] that showed that sulphated-polysaccharides can counteract *N. ceranae*. The contrasting result is probably due to the different origin of the sulphated organic compounds used.

### 4.2. Saccharomyces and Antibiotics

The use of antimicrobial compounds to treat nosemosis was largely studied, being fumagillin the most effective against *N. ceranae* [81]. Its effectiveness was also deeply confirmed in our study, in contrast to Huang et al. [21], also causing low mortality rates. However, its use is controversial, due to its toxicity both to bees and humans, which limits its use in beekeeping because of the presence of possible residues in hive products. Therefore, searching for new antibiotic substances alternative to fumagillin, such as gramicidin and nisin, was one of the aims of this work. The cyclic peptide gramicidin S is produced by *Aneurinibacillus migulanus* DSM2895${}^{T}$, formerly *Bacillus brevis*, that was reported to be highly effective against *N. apis* development [81,82] and active against fungi [83]. Since this substance is no longer available on the market, we opted for gramicidin D (a mixture of gramicidin A, B, and C, produced by *Bacillus brevis* ATCC 8185 [84], a linear channel forming peptides). To the best of our knowledge, gramicidin D has never been tested against microsporidia. The results obtained showed no effects but, on the contrary, an increase in the *N. ceranae* count. A possible explanation may be that gramicidin S compromises the integrity of lipid layer of the cytoplasmic membrane of gram-positive, gram-negative bacteria, and fungi [83], whereas the types A, B, and C are linear peptides with low solubility in water with an antimicrobial action described only against gram-positive bacteria [85]. Nisin, a lantibiotic produced by *Lactococcus lactis*, largely used in food packaging as a preservative and until now never tested as a potential nosemosis treatment, gave different results with respect to gramicidin. This polycyclic polypeptide caused a lower development of *N. ceranae* in honey bee gut, showing a significant effect when the concentration was increased in the derived test and administered to winter honey bees. Based on our results, nisin represents a potential anti-microsporidian for further lab and field tests. Considering the different results obtained to counteract *Nosema* proliferation of the different anti-microbial agents, a higher efficacy of the cyclic molecules with respect to the linear ones may be postulated. This is also confirmed by the work of [36] in which a cyclic surfactin produced by *Bacillus subtilis* was effective against the viability of *Nosema* spores. In the light of these considerations, other experiments are envisaged to validate the hypothesis but also to validate the efficacy of bacteriocin administration in the field. The use of *Saccharomyces* was determined by its presence in nectar and pollen [86,87] and it was also shown to be beneficial to honey bees [88]. The tested concentration (10${}^{11}$ CFU/ml) was found to be toxic to honey bees, whereas the effects on *N. ceranae* were not reproducible between the two performed test. Considering that a significant *N. ceranae* reduction was obtained in the first screening, we believe that the dose of the administered yeast should be adjusted.

### 4.3. Plant Extracts

The administration of plant extracts to honey bees, including extracts from plant material with different extraction methods, essential oils and single main components were widely tested for possible antiparasitic activity against nosemosis with different results [28,30,31,89]. In the case of the natural oils and plant extracts tested in our work, no significant results were obtained against *N. ceranae* development. A possible exception was found when manuka and tea oils in combinations were tested, obtaining a reduction of *Nosema* spores (not statistically significant), thus indicating that a combination of these oils may be a strategy to pursue. Conversely, *Opuntia* extract, notably rich in polyphenols, vitamins, polyunsaturated fatty acids, and amino acids, and whose antimicrobial activity has been studied against *Campylobacter* spp. in poultry [90], did not exhibit any positive effects but led to an increase in the microsporidia development.

Finally, Roussel et al. [80] found positive effects of some sulphated polysaccharides from different marine algae against *N. ceranae* infection. In this study, the Mediterranean seaweed *Padina pavonica* pure extract, possessing antibacterial and anti-*Candida* activities [91], caused a spore load increase in treated honey bees and high toxicity, differently from what expected.

## 5. Conclusions

In this work, we proposed a screening of innovative ingredients that were never tested against the development of *N. ceranae*. Many of these ingredients were selected based on treatments used by beekeepers that often apply some practices basing on empiric knowledge. In the present study it was pointed out that the cyclic antibiotic nisin is efficient in the control of *N. ceranae* even if it shows high mortality rates in cage texts. Moreover, organic acids, such as acetic acid, might be a valid alternative to control the disease avoiding contaminant residuals. It is important to highlight that the efficacy of some compounds seems to be strongly correlated with the seasonal physiology of honey bees, and this factor should be better considered in future studies. Therefore, the promising results from nisin, acetic acid, *p*-coumaric acid, and *Saccharomyces* sp. against the development of nosemosis point out that the use of these ingredients needs to be further explored both in laboratory and field conditions.

## Figures and Tables

**Table 1:** Description of the different active ingredients tested in this work and its relative dose or concentration. ^a^ Dose recommended by the manufacturer. Final concentration of the ingredients are expressed as the amount of ingredient per mL of sugar syrup (1:1 *w*:*v*).

Experimental Theses			Concentration of Ingredient Per Treatment	Reference (When Available)
Ingredient	Treatment Code	Source or Producers		
**Test on Organic Acids**				
**Acetic acid lower concentration**	**AA_L**	Acetic Acid;	84 mM	[40,41]
**Acetic acid higher concentration**	**AA_H**	Merck	0.35 M	[40,41]
**Abscisic acid**	**ABA**	S-(+)-Abscisic Acid Fanda Chem	50 µM	[42]
**p-Coumaric acid**	**pCA**	trans-4-Hydroxycinnamic acid; Merck	31.4 µM	[43]
**Test on** ***Saccharomyces*** **and Antibiotics**				
***Saccharomyces*** **sp. strain KIA1**	**SC**	Isolated by authors from soil	10${}^{11}$ CFU/mL	-
**Gramicidin D lower concentration**	**GRA_L**	Gramicidin from *Bacillus aneurinolyticus*; Merck	7.7 mM	-
**Gramicidin D higher concentration**	**GRA_H**	Gramicidin from *Bacillus aneurinolyticus*; Merck	15.4 mM	-
**Nisin lower concentration**	**NisA_L**	Nisin from *Lactococcus lactis* Merck	7.45 mM	-
**Nisin higher concentration**	**NisA_H**	Nisin from *Lactococcus lactis* Merck	74.5 mM	-
**Test on Wine Derivatives**				
**Ethanol**	**EtOH**	Ethanol; Carlo Erba Reagents	0.69 M	-
**Wine Sulphites (precipitates of potassium pyrosulfite)**	**SPH**	Produced from red wine by a local winemaker and gifted	4 mM	-
**Wine vinegar**	**WA**	Produced from red wine by a local winemaker and gifted	0.3 M	-
**Test on Plant Extracts**				
**Extract of** ***Opuntia ficus-indica***	**OPT**	Produced by authors	0.005 µL/mL	-
**Extract of** ***Padina pavonica***	**PP**	Produced by authors	0.005 µL/mL	-
**Steam distilled Manuka and Tea tree essential oil**	**MT**	Optima Naturalis and ESI s.r.l., respectively	0.75 µL/mL + 0.1 µL/mL	-
**Positive and Negative Controls (included in all tests)**				
**Fumagillin**	**DCH**	Fumagilin-B; Medivet Ltd.	2.59 mM ^a^	Medivet Ltd. guidelines
**Untreated control**	**CTR**	-	-	-

**Table 2:** The table from the first screening (exploratory) and derived tests (confirmatory) are reporting the Log of *N. ceranae* units (NcU) per honey bee gut obtained at 9 days post inoculation with spores for every treatment with dietary ingredients and the relative Standard Deviation. [CTR] = control [***] *p* < 0.01.

Reference Figure	Log NcU ± St.Dev in [CTR]	Experimental Conditions	Log NcU ± St.Dev	*p*-Value
**ORGANIC ACIDS**				
First Test				
2A	7.69 ± 0.24	Acetic Acid_Low [AA_L]	7.57 ± 0.19	
		Abscissic acid [ABA]	7.55 ± 0.21	
		Para-coumaric acid [pCA]	7.69 ± 0.29	
		Fumagillin [DCH]	4.86 ± 0.29	***
Derived Test				
2B	6.64 ± 0.30	Acetic Acid_Low [AA_L]	5.90 ± 1.20	
		Abscissic acid [ABA]	6.86 ± 0.28	
		Para-coumaric acid [pCA]	4.23 ± 0.26	***
		Fumagillin [DCH]	4.11 ± 0.32	***
2C	7.08 ± 1.40	Acetic Acid_High [AA_H]	4.39 ± 1.20	***
		Fumagillin [DCH]	4.27 ± 0.91	***
2D	7.36 ± 0.18	Acetic Acid_High [AA_H]	7.15 ± 0.07	
		Para-coumaric acid [pCA]	7.36 ± 0.36	
		Fumagillin [DCH]	4.72 ± 0.47	***
***Saccharomyces*** **AND ANTIBIOTICS**				
First Test				
2E	7.42 ± 0.24	*Saccharomyces* sp. strain KIA1 [SC]	7.13 ± 0.65	***
		mix, Low concentration [GRA_L]	7.78 ± 0.25	
		Nisin A, Low concentration [NisA_L]	7.53 ± 0.27	
		Fumagillin [DCH]	4.86 ± 0.29	
Derived Test				
2F	7.42 ± 0.24	Gramicidin mix, High concentration [GRA_H]	7.65 ± 0.85	
		Nisin A, High concentration [NisA_H]	6.45 ± 1.48	***
		Nisin A, Low concentration [NisA_L]	7.18 ± 0.99	
		Fumagillin [DCH]	4.51 ± 0.58	***
2G	6.64 ± 0.28	mix, High concentration [GRA_H]	6.67 ± 0.14	
		mix, Low concentration [GRA_L]	6.57 ± 0.30	
		Fumagillin [DCH_A]	4.11 ± 0.32	***
2H	7.36 ± 0.18	*Saccharomyces* sp. strain KIA1 [SC]	7.35 ± 0.09	
		Nisin A, High concentration [NisA_H]	6.99 ± 0.59	
		Fumagillin [DCH]	4.72 ± 0.47	***
**WINE DERIVATES**				
First Test				
3A	7.69 ± 0.24	Ethanol [EtOH]	7.78 ± 0.99	
		Sulphites [SPH]	7.68 ± 0.40	
		Wine vinegar [WA]	7.99 ± 0.24	***
		Fumagillin [DCH]	4.86 ± 0.29	***
Derived Test				
3B	6.64 ± 0.31	Ethanol [EtOH]	6.83 ± 0.18	
		Sulphites [SPH]	6.69 ± 0.16	
		Wine vinegar [WA]	6.67 ± 0.24	
		Fumagillin [DCH]	4.11 ± 0.32	***
**PLANT EXTRACTS**				
First Test				
3C	7.97 ± 0.24	Opuntia ficus-indica extract [OPT]	7.92 ± 0.43	
		Padina pavonica extract [PP]	8.20 ± 0.32	
		Manuka and tea oil [MT]	7.12 ± 1.06	
		Fumagillin [DCH]	5.82 ± 0.20	***
Derived Test				
3D	7.36 ± 0.18	Opuntia ficus-indica extract [OPT]	-	
		Padina pavonica extract [PP]	-	
		Manuka and tea oil [MT]	7.28 ± 0.11	
		Fumagillin [DCH]	4.72 ± 0.47	***

## Data Availability

Elaborated data presented in this study are available on reasonable request from the corresponding author.

## Abbreviations

The following abbreviations are used in this manuscript:

qPCR	Quantitative-polymerase chain reaction
MetAP2	Methionine aminopeptidase-2
AA_L	Acetic acid low concentration
AA_H	Acetic acid high concentration
ABA	Abscissic acid
pCA	Para-coumaric acid
SC	*Saccharomyces* sp. strain KIA1
GRA_L	Mixture of gramicidin A, B, and C at low concentration
GRA_H	Mixture of gramicidin A, B, and C at high concentration
NisA_L	Nisin A at low concentration
NisA_H	Nisin A at high concentration
EtOH	Ethanol
SPH	Sulphites
WA	Wine vinegar
OPT	*Opuntia ficus-indica* extract
PP	*Padina pavonica* extract
MT	Manuka and tea oil mixture
DCH	Fumagillin
CTR	Control
PDB	Potatoes dextrose broth
CFU	Colony forming unit
RH	Relative humidity
PCR	Polymerase chain reaction
ANOVA	Analysis of variance
NcU	*Nosema ceranae* units
